# The association between psychiatric disorders and work-related problems among subway drivers in Korea

**DOI:** 10.1186/s40557-014-0039-7

**Published:** 2014-11-01

**Authors:** Se-eun Kim, Hyoung-Ryoul Kim, Jong-Ik Park, Hae Woo Lee, Jongin Lee, Junsu Byun, Hyeon Woo Yim

**Affiliations:** Department of Occupational and Environmental Medicine, College of Medicine, The Catholic University of Korea, Banpo-daero, Seoul, 137-701 Korea; Department of Psychiatry, School of Medicine, Kangwon National University, Baengnyeong-ro, Chuncheon, 200-722 Korea; Department of Psychiatry, Seoul Medical Center, Sinnae-ro, Seoul, 131-795 Korea; Department of Preventive Medicine and Clinical Research Center for Depression, College of Medicine, The Catholic University of Korea, Banpo-daero, Seoul, 137-701 Korea

**Keywords:** PTSD, Panic disorder, Subway drivers, Korea

## Abstract

**Objectives:**

This study aimed to find the prevalence and occupational risk factors for major psychiatric disorders among subway drivers in South Korea.

**Methods:**

Of all 998 current subway drivers, 995 participated in this study. The Korean version of the Composite International Diagnostic Interview (K-CIDI 2.1) was administered by trained interviewers to diagnose psychiatric disorders in all participants. The questions on socio-demographic characteristics and working conditions included some questions related to a person under train (PUT) experience and work-related problems. One-year prevalence and lifetime prevalence of major depressive disorder (MDD), post-traumatic stress disorder (PTSD), and panic disorder were diagnosed through the interview. The standardized prevalence ratios (SPRs) of these three disorders were calculated in the sample of subway drivers using the 2011 Korean National Epidemiologic Survey data as a basis. Multiple logistic regressions were performed to determine the association between work-related factors and the prevalence of the psychiatric disorders.

**Results:**

The standardized prevalence ratios (SPRs) for a 1-year prevalence of MDD and PTSD among subway drivers were 1.1 (95% CI 0.7-1.7) and 5.6 (95% CI 3.1-8.8), respectively. Conflict with passengers was significantly associated with an increased risk for both MDD and PTSD in 1-year and in lifetime prevalence. Experiencing a sudden stop due to an emergency bell increased the risk of the lifetime prevalence of MDD (OR 2.61, 95% CI 1.14-6.97) and PTSD (OR 7.53, 95% CI 1.77-32.02). The risk of PTSD significantly increased among drivers who once experienced a near accident in terms of both the 1-year prevalence (OR 8.81, 95% CI 1.96-39.3) and the lifetime prevalence (OR 6.36, 95% CI 2.40-16.90).

**Conclusions:**

PTSD and panic disorder were more prevalent among subway drivers than in the general population. We found that having a conflict with passengers, a near accident, and a breakdown while driving can be risk factors for psychiatric disorders among subway drivers. Therefore, a prompt and sensitive approach should be introduced for these high risk groups within the subway company.

## Introduction

Generally, occupational drivers have very stressful jobs because many of them undergo long working times and carry the risk of accident and injury. Furthermore, face-to-face contact with passengers is often needed in their jobs. Many previous studies have addressed the prevalence of psychiatric symptoms and disorders among occupational drivers [1-5], and in particular, subway drivers experience various types of problematic situations during their driving which can be risk factors for psychiatric symptoms or disorders [2,3]. Subway drivers are always at risk of running over a person on the tracks (person under train, PUT) [6-9]. Furthermore, they spend most of their working hours in an underground space, which can cause a sense of isolation and depression [2].

In 2007, a mental health check was conducted targeting subway drivers of a public company in Seoul by using The Korean version of the Composite International Diagnostic Interview (K-CIDI 2.1), a qualified diagnostic tool developed by the World Health Organization (WHO) [10]. The standardized prevalence ratios (SPRs) for the lifetime prevalence of panic disorder and PTSD were 13.3 (95% CI 6.6-22.4) and 2.1 (95% CI 1.1-3.4), respectively. With respect to lifetime prevalence, drivers who experienced PUT showed a significantly higher risk for panic disorder (Odds ratio = 4.2, 95% CI 1.2-16.6) and PTSD (OR = 4.4, 95% CI 1.3-16.4). For the 1-year prevalence, the drivers who experienced PUT had a significantly higher risk for PTSD (OR = 11.7, 95% CI 1.9-225.8). There was no significant value of SPR and OR in major depressive disorder [3].

After the mental health check was conducted, some interventional policies were suggested, which included compulsory physician counseling, minimizing driving hours, protecting drivers from the family of the victims, practicing two-person driving, installing a platform screen door (PSD) and so on [3]. According to the suggestions, most subway stations in Seoul have been equipped with a PSD, and the PSDs have prevented people from falling down on the railway, so PUTs are very rare nowadays [11]. Drivers do not experience PUTs anymore during their driving. However, other policies that were suggested after this survey have not yet been implemented. In particular, one-person driving still continues today although the conversion to two-person driving was one of the key issues [12].

There were several limitations of the survey in 2007. First, the drivers who changed their jobs to other departments did not participate in the survey. Furthermore, some drivers elected not to participate as well. Therefore, the response rate barely reached 86.7% [3]. The company kept a lukewarm attitude and the individuals in charge did not actively support the survey.

Although there has been an effort to improve the mental health of the drivers and their working environment, several cases ended in suicide within the past two years. Consequently, the necessity for active intervention was suggested by performing a mental health survey once again in 2013.

Therefore, this study aims to find the prevalence and occupational risk factors for major psychiatric disorders among subway drivers. Furthermore, we tried to find other factors related to psychiatric disorders different from those of the previous study in 2007.

## Materials and methods

### Study subjects

This study was conducted targeting subway drivers who were employed by a public company in Seoul. Among all 998 current subway drivers, 995 participated in the study (response rate 99.7%). Three drivers were absent from the study due to a sick leave (non-psychiatric diseases), and 15 female drivers were excluded in the analysis. Therefore, 980 male drivers were included in the analysis. All participants were fully informed with respect to the purposes and methods of the study before participating in the interview. Written informed consent was obtained from every subject prior to their participation and this study was approved by the Institutional Review Board of the Seoul St. Mary’s Hospital.

### Assessment of psychiatric disorders

The Korean version of the Composite International Diagnostic Interview (K-CIDI 2.1) was administered by trained interviewers to all participants in order to diagnose psychiatric disorders. The CIDI [13] is a fully structured diagnostic interview that was developed to assess psychiatric diagnoses based on DSM-IV [14]. The K-CIDI was validated by Cho et al. [10] according to the WHO guidelines [15]. The K-CIDI has been used in a previous study performed in the same company in 2007 and in national mental health epidemiological surveys, such as the 2001 Korean Epidemiologic Catchment Area (KECA) [16], 2006 KECA-R [17], and KECA-2011 [18].

Nine trained interviewers carried out face-to-face interviews with all participants. The interviewers were qualified by completing a 5-day training course that included several lectures on general interview skills and on the K-CIDI, mock interviews, and role-playing exercises that followed standard protocols and training materials developed by WHO [19,20].

### Assessment of work-related factors and socio-demographic variables

All participants completed a questionnaire of socio-demographic characteristics and working conditions, which included some questions with respect to PUT experience and work-related problems. The number and time of PUT experiences and the severity of the victim’s injury were asked, and we also inquired about the following work-related problems: conflict with passengers, experience of a sudden stop due to an emergency bell, having a near accident, experience of breakdown, and PUT experience of a colleague. Subway drivers sometimes experience mechanical troubles during their driving (breakdown), and they occasionally suffer from mental distress due to PUT experience of a co-worker although it is not their direct experience [2].

### Data analysis

We examined the 1-year prevalence of MDD, PTSD, and panic disorder. The standardized prevalence ratios (SPRs) of these three disorders in the subway driver sample were calculated based on age-standardized prevalence in the 2011 Korean National Epidemiologic Survey data (KECA-2011) [18]. The SPRs were calculated by dividing the prevalence observed in subway drivers by the expected number from KECA-2011, with a 95% confidence interval (CI). Multiple logistic regressions were performed to determine the association between work-related factors and the prevalence of psychiatric disorders. All analysis was performed with SPSS version 18.0.

## Results

### Prevalence and SPRs of MDD, PTSD, and panic disorder

Table 1 shows the socio-demographic and work-related characteristics of the participants. Most participants were in their 40s and had finished junior college or college. More than half of them had been subway drivers for more than a decade. The 1-year prevalence of MDD, PTSD, and panic disorder were 1.8, 1.6, and 1.0%, respectively. The standardized prevalence ratios (SPRs) for 1-year prevalence of MDD and PTSD were 1.1 (95% CI 0.7-1.7) and 5.6 (95% CI 3.1-8.8) (Table 2). The SPR of panic disorder couldn’t be calculated because the 1-year prevalence of panic disorder among Korean males in 2011 was 0 (zero).

**Table 1:** **Socio-demographic and work-related characteristics of 980 male subway drivers**

**Characteristics**	**Classification**	**N (total = 980)**	**%**
Age, year	20-29	24	2.4
	30-39	193	19.7
	40-49	640	65.3
	≥ 50	123	12.6
Education	High school	178	18.2
	Junior college	308	31.4
	Above college	494	50.4
Marital status	Unmarried	122	12.2
	Married	837	85.4
	Divorced/separated/bereaved	23	2.3
Family income, US dollar/month	< 2,000	5	0.5
	2000-2999	114	11.6
	3000-3999	495	50.5
	4000-4999	221	22.6
	≥ 5000	145	14.8
Years of driving	< 1	40	4.1
	1-4	56	5.7
	5-9	193	19.7
	10-19	575	58.7
	20-29	76	7.8
	≥ 30	40	4.1

**Table 2:** **1-year prevalence and SPRs of MDD, PTSD and panic disorder in 980 subway drivers**

	**1-year prevalence (%)**		
	**SPR (95% CI)**	**Subway drivers (2013)**	**National data (male, 2011)**
MDD	1.1 (0.7-1.7)	1.8	1.8
PTSD	5.6 (3.1-8.8)	1.6	0.2
Panic disorder*	-	1.0	0.0

*The SPR of panic disorder could not be calculated because the 1-year prevalence of panic disorder among Korean males in 2011 was 0 (zero).

### The association between work-related problems and psychiatric disorders

The association between work-related problems and the three psychiatric disorders were determined using a multiple logistic regression. Table 3 shows the risk for the three psychiatric disorders resulting from PUT experience and related factors. For the 1-year prevalence, a higher risk of the three psychiatric disorders was seen among drivers who had experienced PUT, but it wasn’t statistically significant. Likewise, other PUT-related factors were not significantly associated with the risk of three psychiatric disorders. In terms of the lifetime prevalence, PUT experience was marginally associated with a higher risk for PTSD (OR 2.06, 95% CI 0.94-4.55). In particular, the risk for PTSD increased significantly in the group of drivers who had experienced PUT more than two times (OR 3.57, 95% CI 1.32-3.65). The severity of victim’s injury and repeated experience of PUT was not associated with a risk for the three psychiatric disorders.

**Table 3:** **Association between PUT events and related factors and the prevalence of psychiatric disorders**

**PUT experience and related factors**		**1-year prevalence**						**Lifetime prevalence**					
		**MDD**		**PTSD**		**Panic disorder**		**MDD**		**PTSD**		**Panic disorder**	
		**OR**	**95% CI**	**OR**	**95% CI**	**OR**	**95% CI**	**OR**	**95% CI**	**OR**	**95% CI**	**OR**	**95% CI**
PUT experience (Ref = no)	Yes	1.99	0.72-5.53	1.54	0.52-4.55	1.31	0.31-5.50	1.13	0.55-2.31	2.06	0.94-4.55	1.75	0.59-5.14
Number of PUT experience (Ref = 0)	1	2.01	0.66-6.11	1.77	0.31-4.47	1.83	0.45-7.50	0.95	0.40-2.25	1.45	0.55-3.85	1.67	0.50-5.58
	≥2	1.94	0.39-9.62	2.36	0.57-9.70	0	0	1.56	0.56-4.34	3.57	1.32-3.65	1.95	0.39-9.79
Severity of victim’s injury (Ref = alive)	Death	0.59	0.13-2.72	2.49	0.27-23.27	0.4	0.04-4.55	0.79	0.25-2.53	1.39	0.40-4.82	0.39	0.07-2.18
When PUT was experienced (Ref = over 5 years)	Within 5 years	1.04	0.77-1.05	1.01	0.11-9.06	1.04	0.07-15.38	0.47	0.09-2.46	0.33	0.03-2.63	0.46	0.05-4.68

Note: Odds ratio and 95% confidence interval were estimated by performing multiple logistic regression, adjusted for age. Except for age, other socio-demographic factors were not associated with psychiatric disorders in univariate analysis.

The association between work-related problems and the prevalence of psychiatric disorders is presented in Table 4. Conflict with passengers was significantly associated with the risk of both MDD and PTSD in 1-year and in lifetime prevalence, but it was not associated with the risk for panic disorder. A sudden stop due to an emergency bell increased the risk of the lifetime prevalence of MDD (OR 2.61, 95% CI 1.14-6.97) and PTSD (OR 7.53, 95% CI 1.77-32.02), and the risk for PTSD significantly increased among drivers who had once experienced a near accident for both the 1-year prevalence (OR 8.81, 95% CI 1.96-39.3) and the lifetime prevalence (OR 6.36, 95% CI 2.40-16.90). Experience of breakdown was not significantly associated with three psychiatric disorders. The study results showed that PUT experience of a colleague increased the risk of lifetime prevalence of PTSD (OR 2.84, 95% CI 1.32-6.12) and the risk of MDD and panic disorder for both the 1-year prevalence (MDD: OR 4.54, 95% CI 1.76-11.70, panic disorder: OR 3.54, 95% CI 1.01-12.38) and the lifetime prevalence (MDD: OR 2.92, 95% CI 1.54-5.54, panic disorder: OR 2.78, 95% CI 1.02-7.58).

**Table 4:** **Association between the prevalence of psychiatric disorders and work-related problems during the past year**

	**1-year prevalence**						**Lifetime prevalence**					
**Work-related problem**	**MDD**		**PTSD**		**Panic disorder**		**MDD**		**PTSD**		**Panic disorder**	
	**OR**	**95% CI**	**OR**	**95% CI**	**OR**	**95% CI**	**OR**	**95% CI**	**OR**	**95% CI**	**OR**	**95% CI**
Conflict with passengers	3.13	1.21-8.11	3.21	1.14-9.03	0.57	0.12-2.76	2.02	1.07-3.85	3.32	1.55-7.12	1.11	0.38-3.26
Sudden stop due to an emergency bell	4.17	0.95-18.3	3.66	0.82-16.4	2.04	0.43-9.69	2.61	1.14-5.97	7.53	1.77-32.02	1.55	0.50-4.87
Near accident	1.01	0.40-2.58	8.81	1.96-39.3	1.26	0.36-4.39	1.35	0.72-2.54	6.36	2.40-16.90	1.27	0.47-3.43
Breakdown	0.81	0.18-3.57	1.71	0.48-6.14	0.71	0.09-5.71	1.14	0.47-2.77	1.89	0.75-4.75	0.43	0.06-3.30
PUT experience of a colleague	4.54	1.76-11.70	0.55	0.12-2.47	3.54	1.01-12.38	2.92	1.54-5.54	2.84	1.32-6.12	2.78	1.02-7.58

Note: Odds ratio and 95% confidence interval were estimated by performing multiple logistic regression, adjusted for age. Except for age, other socio-demographic factors were not associated with psychiatric disorders in univariate analysis.

## Discussion

This study shows that there is a higher prevalence of the three psychiatric disorders among subway drivers than that in the national data. In KECA-2011, the 1-year prevalence of MDD, PTSD, and panic disorder among adult males were 1.8%, 0.2%, and 0.0%, respectively [18]. Based on the KECA-2011 data, the SPRs of MDD and PTSD were 1.1 (95% CI 0.7-1.7) and 5.6 (95% CI 3.1-8.8), respectively. Therefore, the prevalence of MDD seems to be similar to that of the general population. On the other hand, the SPR of PTSD was 5.6. It means that the risk of PTSD among subway drivers was 5.6 times higher than that in the general population. Due to the healthy worker effect, psychiatric disorders are less prevalent in the working population. However, in this study, subway drivers easily have PTSD and panic disorder. The working environment might be a cause of a higher prevalence of psychiatric disorders in this group. Subway drivers have potential exposure to PUT events when driving their trains [3-9]. However, after 2007, PSDs were established in most stations in Seoul. Nowadays, there are very few PUT cases in a year. Therefore, other work-related factors, such as general work stress, hierarchical culture, organizational justice, and conflict with passengers can be causes for newly developed psychiatric illness in this group.

In the study performed in 2007 in the same company, the 1-year prevalence of MDD, PTSD, and panic disorder were 1.3%, 0.9% and 0.6%, respectively. In the present study, the 1-year prevalence of MDD, PTSD and panic disorder were 1.8%, 1.6% and 1.0%, respectively. These results show that the 1-year prevalence of the three psychiatric disorders increased relative to those found in the previous study. There can be several reasons for that. One of them might be that there was a higher response rate compared to the previous study (99.6% vs 86.7%) [3]. It means that an exclusion of a high risk group could have occurred in the previous study. Actually, 4 drivers who did not participate in the previous study (in 2007) were recognized to have a psychiatric disorder in the present study. Second, other newly developed stressful conditions, such as an increase of conflict with passengers, operating new train systems, and conflicts among workers due to a plurality of labor unions, emerged in their workplace after 2007. Third, any other support systems for high risk group were not introduced in this company in spite of researchers’ recommendations in 2007 (two-driver system, supporting drivers who have experienced PUTs, etc.).

As mentioned above, the result of this study does not show a significant association between psychiatric disorders and PUT related factors, with the exception of the association between the number of PUT experiences and the lifetime prevalence of PTSD. Other work-related problems, on the other hand, are significantly associated with the prevalence of the psychiatric disorders. Two subway companies that the Seoul City government runs equipped most stations with PSDs. After that, the incidence of PUTs drastically decreased; thus, PUTs occurs less than twice a year. Therefore, other work-related problems have become more prominent in recent years, including conflicts with passengers, sudden stops due to an emergency bell, near accidents, breakdown, and PUT experience of a colleague. These stressful events derived from a qualitative study with drivers were asked. Although these problems seem to be less serious than PUT, they appear to elevate the risk of three psychiatric disorders in subway drivers.

The researchers of this study recommended the company to perform mental health exams regularly. It is expected that the high-risk group and the individuals that present symptoms will be found earlier and can be treated properly. As a result of a proposal by the researchers, in the late 2013 the company established a counseling center for employees where a doctor and a psychological counselor are available. There are three categories of interventions that can solve mental health problems among subway drivers. First is the common approach targeting all subway drivers. This includes various policies to enhance the mental health or to change perceptions. Second, active surveillance is needed for a high-risk group, for example, drivers who experienced PUT or suffered seriously from a conflict with a passenger. This aims to support vulnerable workers in order to overcome mental problems at an early stage. Third is the early intervention for drivers who show symptoms in order to prevent the exacerbation of disease and to facilitate a return-to-work process. Likewise, multi-dimensional approach seems to be needed to improve the mental health of subway drivers.

This study has several limitations. First, in this study, we could not identify drivers at a pre-disease stage. Even though CIDI was used to define psychiatric disorders, a screening tool for early detection and prevention should also be applied simultaneously or step by step. Second, we did not include other possible related disorders as outcome variables (e.g., somatoform disorders, general anxiety disorders, and substance-related disorders). Finally, a lower prevalence of psychiatric disorders resulted in larger CIs, meaning that the results of this study can vary as a result of a variation due to one or two cases. However, we used CIDI, where the target disease can be diagnosed correctly, but the survey results in a low prevalence of mental disorders.

In this study, PTSD and panic disorders were still more prevalent among subway drivers in comparison with the general population. We found that conflict with passengers, a near miss accident and a breakdown while driving can be risk factors for psychiatric disorders among subway drivers. Therefore, a prompt and sensitive approach for these high risk groups should be introduced in this company.

## Conclusions

PTSD and panic disorder were more prevalent among subway drivers than in the general population. We found that having a conflict with passengers, a near accident, and a breakdown while driving can be risk factors for psychiatric disorders among subway drivers. Therefore, a prompt and sensitive approach should be introduced for these high risk groups within the subway company.

## References

[1] Choi WS, Cho SA, Cho YS, Koo JW, Kim KY, Kim H-R (2011). The relationship between the experience of an accident and post traumatic stress disorder in bus drivers. Korean J Occup Environ Med.

[2] Jo SJ, Yim HW, Kim HR, Lee KS, Park JI, Chang SM (2010). Association of subway driver’s depressive symptoms and experience of work-related problems. Epidemiol Health.

[3] Kim HR, Yim HW, Jo SJ, Choi B, Jeong SH, Lee KS, Park JI, Chang SM (2013). Major depressive disorder, panic disorder, and post-traumatic stress disorder in Korean subway drivers. Int Arch Occup Environ Health.

[4] Woo JM, Kang TY, Lee JE (2005). Increasing risk of mental health problems among subway drivers experiencing accidents on the track. Korean J Occup Environ Med.

[5] Yum BS, Roh JH, Ryu JC, Won JU, Kim CN, Lee JE, Kim KY (2006). Symptoms of PTSD according to individual and work environment characteristics of Korean railroad drivers with experience of person-under-train accidents. J Psychosom Res.

[6] Cothereau C (2004). Professional and medical outcomes for French train drivers after “person under train” accidents: three year follow up study. Occup Environ Med.

[7] Karlehagen S, Malt UF, Hoff H, Tibell E, Herrstromer U, Hildingson K, Leymann H (1993). The effect of major railway accidents on the psychological health of train drivers—II. A longitudinal study of the one-year outcome after the accident. J Psychosom Res.

[8] Theorell T, Leymann H, Jodko M, Konarski K, Norbeck HE (1994). Person under train’ incidents from the subway driver’s point of view—A prospective 1-year follow-up study: the design, and medical and psychiatric data. Soc Sci Med.

[9] Vatshelle Å, Moen BE (1997). Serious on-the-track accidents experienced by train drivers: psychological reactions and long-term health effects. J Psychosom Res.

[10] Cho MJ, Hahm BJ, Suh DW, Hong JP, Bae JN, Kim JK, Lee DW, Cho SJ (2002). Development of a Korean Version of the Composite International Diagnostic Interview (K-CIDI). J Korean Neuropsychiatr Assoc.

[11] Bahk EJ (2013). Subway Screen Doors to be Mandatory. The Korea Times.

[12] Lee HS (2012). Subway Engineers Vulnerable to Panic Disorder. The Korea Times.

[13] WHO (1990). Composite International Diagnostic Interview (CIDI) Version 1.0.

[14] American Psychiatric Association (1994). Diagnostic and Statistical Manual of Mental Disorders.

[15] WHO (1997). Procedures for the Development of New Language Versions of the WHO Composite International Diagnostic Interview (WHO-CIDI).

[16] Cho MJ, Hahm BJ, Kim JK, Park KK, Chung EK, Suh TW, Kim SU, Cho SJ, Lee JY, Hong JP (2004). Korean Epidemiologic Catchment Area (KECA) study for psychiatric disorders: prevalence of specific psychiatric disorders. J Korean Neuropsychiatr Assoc.

[17] Cho MJ, Chang SM, Hahm BJ, Chung IW, Bae A, Lee YM, Ahn JH, Won SH, Son J, Hong JP (2009). Prevalence and correlates of major mental disorders among Korean adults: a 2006 National Epidemiologic Survey. J Korean Neuropsychiatr Assoc.

[18] *The Epidemiological Survey of Mental Disorders in Korea.* Seoul: Ministry of Health and Welfare; 2011.

[19] WHO (1997). CIDI, Core Version 2.1 Interview’s Manual.

[20] WHO (1997). CIDI, Core Version 2.1 Trainer’s Manual.

